# Longitudinal assessment of anxiety and depression symptoms in U.S. adolescents across six months of the coronavirus pandemic

**DOI:** 10.1186/s40359-022-01028-8

**Published:** 2022-12-29

**Authors:** Anne E. Bowen, Katherine L. Wesley, Emily H. Cooper, Maxene Meier, Jill L. Kaar, Stacey L. Simon

**Affiliations:** 1 Division of Pulmonary and Sleep Medicine, Children’s Hospital Colorado, 13123 E 16th Ave, B395, Aurora, CO 80045 USA; 2 Department of Pediatrics, University of Colorado School of Medicine, 13123 E 16th Ave, Aurora, CO USA; 3 Center for Research in Outcomes for Children’s Surgery, University of Colorado School of Medicine, 13123 E 16th Ave, Aurora, CO USA

**Keywords:** Depression, Anxiety, Adolescence, Negative affect, Mental health

## Abstract

**Background:**

The present study aimed to describe anxiety and depression symptoms at two timepoints during the coronavirus pandemic and evaluate demographic predictors.

**Methods:**

U.S. high school students 13–19 years old completed a self-report online survey in May 2020 and November 2020-January 2021. The Patient-Reported Outcomes Measurement Information System (PROMIS) Pediatric Depression and Anxiety short forms queried depression and anxiety symptoms.

**Results:**

The final sample consisted of 694 participants (87% White, 67% female, 16.2 ± 1.1 years). Nearly 40% of participants reported a pre-pandemic depression diagnosis and 49% reported a pre-pandemic anxiety diagnosis. Negative affect, defined as both moderate to severe depression and anxiety PROMIS scores, was found in ~ 45% of participants at both timepoints. Female and other gender identities and higher community distress score were associated with more depression and anxiety symptoms. Depression symptoms T-score decreased slightly (− 1.3, *p*-value  ≤ 0.001).

**Conclusion:**

Adolescent mental health screening and treatment should be a priority as the pandemic continues to impact the lives of youth.

**Supplementary Information:**

The online version contains supplementary material available at 10.1186/s40359-022-01028-8.

## Background

Mental health of adolescents in the U.S. is of significant concern. A national survey of youth ages 8–15 years old found that 13% of youth experienced a mental health disorder, including anxiety disorders and depression [21]. Frequently, mental health disorders persist into adulthood if left untreated, therefore, early detection is critical [16]. Mental health symptoms may be increasing; numerous studies have reported an increased need for mental health services for youth, including a 50% increase in inpatient mental health admissions between 2006 and 2011 [1, 28, 36]. Demographic factors, such as gender, socioeconomic status (SES), and race/ethnicity are associated with depression and anxiety symptoms. In adolescence, girls tend to have higher rates of depression than boys [37]. Lower SES is related to higher rates of anxiety and depression symptoms in children and adolescents [17].

The coronavirus disease 2019 (COVID-19) pandemic may have further impacted mental health of youth. In spring of 2020, isolation guidelines were imposed in the U.S. to mitigate the spread of the emerging COVID-19, including closure of non-essential businesses, closure of schools and universities, and limitations on private and public gatherings [11]. These restrictions impacted typical activities of adolescents, including how they attended school and work, participation in and access to sports and other extracurricular activities, and interactions with peers. While these isolation policies were imposed to combat the spread of the virus, they may have caused unintended impacts on mental health.

Emerging evidence from international studies suggest depression and anxiety symptoms in adolescents during the early COVID-19 pandemic were high. Additionally, research investigating specific risk factors for adolescents during the pandemic suggest that race/ethnicity and gender identity may impact risk for mental health problems [13, 19, 32]. In a sample of adolescents and young adults (age 14–35 years) in China, 40% reported psychological distress (anxiety and depression symptoms) and 14% reported PTSD symptoms via online survey two weeks after the World Health Organization declared COVID-19 a public health emergency [18]. In another online survey, 45% of a college student sample in China reported psychological distress, defined as a score above 19 on the Kessler Psychological Distress Scale which measures anxiety and depression symptoms, during the initial COVID-19 outbreak (January 2020) [10]. Several online surveys of children and adolescents in China examined factors associated with anxiety and depression during the pandemic. Female gender, adolescent age (compared to child age), residing in an urban area, emotion-focused coping, and low social support correlated with higher anxiety symptoms [6, 30]. Low social support and smartphone and internet addiction were associated with higher depression symptoms [6, 30]. In contrast, an online survey of children and adolescents (grades 1–12) conducted in March of 2020 found that parent-child discussions about COVID-19 correlated with higher life satisfaction and lower depression and anxiety symptoms [34].

In a sample of 16–25 year old individuals living in Germany and using a digital mental health application, 38% met criteria for psychological distress (anxiety and depression symptoms) on the Kessler Psychological Distress Scale during May 2020 [32]. McElroy et al. 20 created a Pandemic Anxiety Scale (PAS) to capture anxiety unique to the pandemic, utilized in a sample of UK children and adolescents (age 4–16 years). In this sample, lower household income, female gender, and having a chronic physical health problem were associated with higher pandemic anxiety as measured by the PAS. In a sample of Canadian adolescents who completed an online survey in April of 2020, adolescents reported high concern of the pandemic and high pandemic-related stress. Additionally, higher hours spent connecting with friends virtually correlated with higher depressive symptoms, while physical activity correlated with lower depression. Further, female gender was a predictor of depressive symptoms [8].

Two longitudinal studies examined anxiety and depression in adolescents prior to and during the COVID-19 pandemic. Magson et al. conducted a study which surveyed 248 adolescents living in an urban area of Australia at two timepoints: 12 months prior to the pandemic (T1), and two months following initial pandemic lockdown (T2). Adolescents reported higher depressive symptoms and anxiety at T2 compared to T1, as measured on the Short Mood and Feelings Questionnaire- Child Version and Generalized Anxiety subscale of the Spence Children’s Anxiety Scale, respectively. Female gender, worries about COVID-19, and difficulties with online learning predicted increases in anxiety and depressive symptoms from T1 to T2 [19]. In a sample of 322 U.S adolescents surveyed January of 2020 (prior to the pandemic in the U.S.) and at 3 timepoints after the implementation of COVID-19 isolation orders (mid-April 2020, early May 2020, and late May 2020), internalizing problems decreased from prior to the pandemic to the first follow-up, particularly for youth who reported high levels of internalizing problems prior to the pandemic [27]. No studies, to our knowledge, have examined anxiety and depression in adolescents during the COVID-19 pandemic longitudinally across a longer time period.

A significant limitation to existing studies conducted during the pandemic is that many adolescent studies also included either young adults up to age 35 years or younger children [18, 32, 34]. Adolescents may have different experiences than young adults, and research on exclusively adolescent samples is necessary to understand their unique pandemic experiences. Many published studies are based in China and Europe, however, there may be cultural differences in both mental health and pandemic-related anxieties, therefore, U.S.-based studies are important to inform clinicians in the U.S. Further, at the time of the implementation of our study, research focused primarily on the initial wave of the pandemic and did not investigate mental health over time. This is critical given the ongoing status of the COVID-19 pandemic and continued after effects; mental health status may shift over the course of the pandemic and aftermath.

The goal of the present study was to describe anxiety and depression symptoms in U.S. high school students during the early COVID-19 pandemic and evaluate potential demographic predictors (race, ethnicity, gender, and community distress score (a surrogate measure of SES)). Further, we aimed to investigate longitudinal changes in mental health between the beginning of the pandemic and a 6-month follow-up. We hypothesized that racially and ethnically minoritized individuals, those living in areas with higher community distress, and females would have elevated symptoms of anxiety and depression compared to other groups. Given prior research showing that, if left untreated, anxiety and depression worsen over time, we also hypothesized that over the course of 6 months of the pandemic depression and anxiety symptoms would increase, and that disparities by gender, race, and community distress may be exacerbated [9, 16, 22].

## Methods

### Participants

An online self-report survey was advertised nationally to adolescents via Facebook newsfeed during the early COVID-19 pandemic (see Additional file 1). The advertisement linked directly to the study description and consent on the University of Colorado Denver Research Electronic Data Capture (REDCap) platform. Respondents who provided consent (if age 18 or older) or assent (if under 18 years old, with indication of parental consent), were 13–19 years old, enrolled in high school at time of initial survey, lived in the U.S., and had complete data for the variables of interest (age, gender, race, ethnicity, zip code, and PROMIS depression and anxiety short forms) were included in analyses. Timepoint 1 (T1) survey responses were collected from May 5, 2020 to May 18, 2020. Participants were asked to provide an email address at the end of the survey if they were willing to participate in a follow-up survey at timepoint 2 (T2). T2 survey links were sent to provided emails in November 2020. Responses were collected from participants between November 24, 2020 and January 13, 2021. Participants did not receive compensation for participating in the survey. The study was approved by the Colorado Multiple Institutional Review Board.

### Demographics

Participants were asked to indicate their age, school grade, race(s), ethnicity, and gender (participants chose from male, female, non-binary, or other, and responses were categorized into male, female, and other), and report if they had previously received a diagnosis of anxiety or depression from a health care professional. To assess SES, zip codes provided by respondents were assigned a continuous distress score from 0 to 100 with higher scores indicating greater distress, according to the Economic Innovation Group’s Distressed Communities Index (DCI). Continuous scores were then categorized by the DCI into the following groups: Prosperous, Comfortable, Mid-tier, At Risk, Distressed. The DCI indicates economic well-being of the zip code based on poverty rate, education level, employment status, median household income, and change in employment and establishments [7].

### Mood

The Patient-Reported Outcomes Measurement Information System (PROMIS) Pediatric Depression and PROMIS Pediatric Anxiety v2.0 short forms were used to assess mood symptoms at T1 and T2. The 8-item questionnaires ask participants to rate frequency of symptoms in the past 7 days on a 5-point Likert-type scale from “1 = Never” to “5 = Almost Always”. Raw scores range from 8 to 40 with higher scores indicating more severe symptoms. Raw scores were converted to T-scores using the Health Measures Scoring Service [24]. These measures have been validated within clinical and community adolescent samples [15]. For both measures, T-scores were classified as follows: <55 = normal, ≥ 55 and < 60 = mild, ≥ 60 and < 70 = moderate, ≥ 70 = severe [25]. Negative affect was defined as a score of moderate to severe on both PROMIS anxiety and depression measures.

### Statistical analysis

Demographic and clinically relevant variables are summarized using mean with standard deviation (SD) for continuous variables and frequency with percentage for categorical variables. Paired T-tests examined change in T-scores for depression and anxiety from T1 to T2. McNemar’s Chi-squared test was used to compare change in categorical variables from T1 to T2. Six multivariable linear regression models examined predictors of PROMIS depression or anxiety T-scores at T1, T2, and for the change from T1 to T2. Multivariable logistic regression models examined predictors of negative affect at T1 and T2. All models adjusted for gender (categorized as male, female, or other), age, community distress score, race (categorized as White vs. non-White), and ethnicity. Additionally, T2 models and change from T1 to T2 models also adjusted for T1 scores of the respective outcome. Sensitivity analyses were performed to determine if those that met inclusion criteria at T1 differed from those that met inclusion criteria at both T1 and T2. Descriptive statistics of T1 demographics and PROMIS depression and anxiety scores were compared using t-tests and chi-squared tests or Fisher’s exact tests. Additionally, all T1 regression models were refit including everyone who met inclusion criteria at T1, and results were assessed for changes in statistical and clinical significance. Sensitivity analyses of T2 regressions could not be performed due to the paired nature of the statistical methods used. All analyses were conducted using R language and environment for statistical computing (Vienna, Austria) [31]. A *p*-value threshold of 0.05 was used to determine statistical significance.

## Results

Of the 7186 individuals that initially clicked on the survey link, 2947 met inclusion criteria and consented/assented at T1. Of those participants, 1649 provided an email and consented to be contacted for follow-up, and 694 consented/assented, met inclusion criteria and had complete data at both T1 and T2. We compared the full sample of 2947 participants to the sample of 694 participants and found no significant differences in characteristics nor outcomes of interest, except for a 3.5 point difference in community distress score (on a scale of 0–100) and index (*p* < 0.001 and *p*  = 0.005, respectively). Furthermore, when refitting all T1 models to include everyone with T1 data, the statistical and clinical significance of the regression results remained except as noted below (see Additional files 2, 3: Tables S1, S2). Therefore, for the purposes of these analyses, we have only included participants with a complete data set at T1 and T2 (n = 694).

This sample was 66.7% female, 87.0% White, 5.48% Hispanic, and on average 16.20 ± 1.06 years old. Based on the DCI, 23.8% of the sample lived in at-risk or distressed communities. 40% of the sample reported a pre-pandemic diagnosis of depression, and 49.0% reported a pre-pandemic diagnosis of anxiety. Full demographic characteristics are presented in Table 1.

**Table 1:** Demographics at T1

	Total sample (N = 694)
Age (years)	16.20 (1.06)
Gender	
Female	463 (66.7%)
Male	186 (26.8%)
Other	45 (6.48%)
Race	
White	604 (87.0%)
Black	13 (1.9%)
Asian	15 (2.2%)
American Indian or Alaska Native	10 (1.4%)
Native Hawaiian or Pacific Islander	0 (0.0%)
More than One Race	43 (6.2%)
Other	9 (1.3%)
Ethnicity	
Hispanic	38 (5.5%)
Non-Hispanic	656 (94.5%)
Distressed Community Index	35.50 (28.20)
Prosperous	265 (38.2%)
Comfortable	156 (22.5%)
Mid-Tier	108 (15.6%)
At-Risk	106 (15.3%)
Distressed	59 (8.5%)
Reported Depression Diagnosis	276 (39.8%)
Reported Anxiety Diagnosis	340 (49.0%)

Data are shown as mean (standard deviation) or frequency (percentage)

Data on anxiety and depression symptom scores and severity across both timepoints are presented in Table 2. At T1 48.0% endorsed moderate to severe anxiety and 72.3% endorsed moderate to severe depression on the PROMIS scales. At T2, 50.6% endorsed moderate to severe anxiety and 70.6% endorsed moderate to severe depression. Negative affect was found in 45.4% of participants at T1 and 45.8% of participants at T2 (Table 2).

**Table 2:** PROMIS Anxiety and Depression at T1 and T2

	T1	T2	Change (95% CI)	*p*-value
PROMIS Anxiety T-score	58.8 (13.3)	59.1 (12.1)	0.26 (0.22, 0.30)	0.539
PROMIS Anxiety Categorical				0.061
Normal	255 (36.7%)	228 (32.9%)		
Mild	106 (15.3%)	115 (16.6%)		
Moderate	193 (27.8%)	229 (33.0%)		
Severe	140 (20.2%)	122 (17.6%)		
PROMIS Depression T-score	65.3 (11.1)	64.0 (10.5)	− 1.30 (− 1.34, − 1.27)	**< 0.001**
PROMIS Depression Categorical				**0.015**
Normal	112 (16.1%)	112 (16.1%)		
Mild	80 (11.5%)	92 (13.3%)		
Moderate	272 (39.2%)	303 (43.7%)		
Severe	230 (33.1%)	187 (26.9%)		
Negative affect	315 (45.4%)	318 (45.8%)		0.881

Data are shown as mean (standard deviation) or frequency (percentage). PROMIS measures T-scores are determined as follows: T-scores with a mean of 50 and SD of 10, T-scores < 55 classified as normal, T-scores between 55 and 60 classified as mild, T-scores between 60 and 70 classified as moderate, T-scores over 70 classified as severe. A p value of < 0.05 was determined to be significant (in bold)

At T1, relative to males, female and other gender identities were associated with higher depression (*p* < 0.001 for both) and anxiety (p*p*< 0.001 and *p* = 0.002, respectively) T-scores. Higher community distress score was also associated with higher depression (*p* = 0.003) and anxiety (*p*  = 0.001) T-scores. Race, ethnicity, and age were not significantly associated with anxiety T-score or depression T-score at T1 (Table 3).

**Table 3:** Predictors of PROMIS depression and anxiety T-scores at T1

Predictors	PROMIS depression T-score T1			PROMIS anxiety T-score T1		
	Estimates	95% CI	*P*	Estimates	95% CI	*P*
(Intercept)	53.11	40.28–65.95	**< 0.001**	47.65	32.23–63.07	**< 0.001**
Gender: Female vs. Male	4.67	2.82–6.52	**< 0.001**	6.02	3.80–8.24	**< 0.001**
Gender: Other vs. Male	6.44	2.89–9.99	**< 0.001**	6.71	2.44–10.97	**0.002**
Race: White vs. Non-White	0.58	− 1.84–3.01	0.637	0.90	− 2.01–3.81	0.544
Ethnicity: Hispanic vs. Non-Hispanic	0.89	− 2.66–4.44	0.623	0.61	− 3.66–4.88	0.779
Age	0.41	− 0.36–1.17	0.296	0.24	− 0.67–1.16	0.602
Distress Score (Continuous)	0.04	0.01–0.07	**0.003**	0.06	0.02–0.09	**0.001**

A *p*-value of < 0.05 was determined to be significant (in bold)

Controlling for T1 depression and anxiety T-scores, gender identity other than male or female was associated with higher depression (*p* = 0.047) and anxiety (*p* = 0.034) T-scores at T2 when compared to males (Table 4). Compared to males, female identity was associated with increased odds of negative affect at T1 (*p* < 0.001). In the full sample of respondents at T1 (n = 2947), gender identity other than male or female was also associated with increased odds of negative affect at T1 (*p* < 0.001) (see Additional file 4: Table S3). Community distress score was associated with affect at both T1 and T2 (*p* = 0.017 and *p* = 0.020, respectively). In the sample of all respondents at T1, distress score is not significantly associated with affect (*p* = 0.076) (see Additional file 4: Table S3). Controlling for depression T-score at T1, age was associated with higher depression T-score at T2 (*p*  = 0.043). Race and ethnicity were not significantly associated with any outcome—depression symptoms, anxiety symptoms, or negative affect—at T1 or T2 (Tables 4 and 5).

**Table 4:** Predictors of PROMIS depression and anxiety T-scores at T2

Predictors	PROMIS depression T-score T2			PROMIS anxiety T-score T2		
	Estimates	95% CI	*p*	Estimates	95% CI	*p*
(Intercept)	34.95	24.74–45.15	**< 0.001**	28.88	17.28–40.49	**< 0.001**
T-score at T1	0.58	0.53–0.64	**< 0.001**	0.55	0.49–0.60	**< 0.001**
Gender: Female vs. Male	0.44	− 0.99–1.87	0.548	1.33	− 0.33–2.99	0.117
Gender: Other vs. Male	2.76	0.04–5.48	**0.047**	3.40	0.25–6.55	**0.034**
Race: White vs. Non-White	− 0.06	− 1.90–1.77	0.945	-0.03	− 2.16–2.11	0.981
Ethnicity: Hispanic vs. Non-Hispanic	0.40	− 2.29–3.10	0.769	0.63	− 2.50–3.76	0.691
Age	− 0.60	− 1.18 to − 0.02	**0.043**	-0.22	− 0.90–0.45	0.512
Distress Score (Continuous)	0.01	− 0.01–0.03	0.529	0.01	− 0.01–0.04	0.293

A *p*-value of < 0.05 was determined to be significant (in bold)

**Table 5:** Predictors of negative affect at T1 and T2

Predictors	Negative affect T1			Negative affect T2		
	Odds Ratios	95% CI	*p*	Odds Ratios	95% CI	*p*
(Intercept)	0.31	0.03–3.48	0.344	0.21	0.01–3.14	0.258
Negative affect at T1				7.84	5.57–11.13	**< 0.001**
Gender: Female vs. Male	2.13	1.49–3.07	**< 0.001**	1.34	0.90–2.00	0.156
Gender: Other Gender vs. Male	1.80	0.92–3.52	0.084	2.31	1.08–4.96	**0.030**
Race: White vs. Non-White	1.04	0.66–1.65	0.855	1.13	0.67–1.89	0.650
Ethnicity: Hispanic vs. Non-Hispanic	1.43	0.73–2.82	0.295	1.26	0.59–2.69	0.551
Age	1.01	0.87–1.16	0.906	0.99	0.84–1.16	0.892
Distress Score (Continuous)	1.01	1.00–1.01	**0.017**	1.01	1.00–1.01	**0.020**

A *p*-value of < 0.05 was determined to be significant (in bold)

PROMIS depression T-scores decreased by an average of -1.30 points from T1 to T2 (*p* < 0.001). There were no statistically significant changes in PROMIS anxiety score or percentage of those with negative affect from T1 to T2.

## Discussion

In this sample of U.S. adolescents assessed at two timepoints during the COVID-19 pandemic, 40% of participants reported a pre-pandemic diagnosis of depression and 49% reported a pre-pandemic diagnosis of anxiety. Comparatively, the National Institute of Mental Health reports a 13.3% prevalence of Major Depressive Disorder and 31.9% prevalence of anxiety disorders in adolescents age 12–17 years [23]. During COVID-19, nearly half of participants endorsed moderate to severe anxiety at both timepoints and nearly three quarters endorsed moderate to severe depression at both timepoints as measured by the PROMIS scales. These findings are similar to existing studies of mental health symptoms in adolescents during the COVID-19 pandemic: Liang et al., Gong et al., and Rauschenberg et al. all found high rates (ranging from 38 to 45%) of psychological distress (anxiety and depression symptoms) in adolescents and young adults living in China and Germany during the COVID-19 pandemic [10, 18, 32].

The high rates of anxiety and depression symptoms seen in our population may be a result of prior conditions, given reported high rates of pre-pandemic anxiety and depression, and not a reflection on the impact of the pandemic on adolescent mental health. Onset of psychiatric illnesses is most common during early adolescence [26]. Without treatment, mental illness often persists into adulthood [16]. Therefore, evaluation, prevention, and treatment of mental health symptoms is critical during this developmental period to ensure that adolescents have a successful transition to adulthood [26]. Future research should examine if there are changes in population level anxiety and depression diagnoses as we continue to deal with the aftereffects of the COVID-19 pandemic. Further, research should examine how anxiety and depression symptoms of those with a pre-pandemic anxiety or depression diagnosis changed during the pandemic.

Our results align with research prior to COVID-19 that found females and gender-diverse adolescents are at a greater risk for psychopathology during adolescence than their male peers [4, 5, 37]. Emerging evidence shows that mental health symptoms of gender diverse youth are higher than those of their peers [13]. However, the portion of those identifying as a gender other than male or female in our sample was low (6.5%), and we did not assess changes in anxiety and depression symptoms from prior to the pandemic. Therefore, future research should continue to explore mood in gender diverse youth as communities continue to deal with challenges related to the COVID-19 pandemic as this group is already at high risk of mental health symptoms.

Contrary to our hypotheses, neither race nor ethnicity were associated with anxiety and depression symptoms. This was unexpected as, compared to White youth, Black and Hispanic/Latino youth are more likely to be exposed to social risk factors of mental health disorders, such as poverty, community and domestic violence, and social isolation [2, 3]. The low percentage of racially minoritized adolescents in our sample did not allow us to distinguish racial categories further than “White” and “Non-White” for statistical analyses, which may have affected these findings. Future research should strive to include a larger percentage of racially and ethnically minoritized youth to define racial categories separately for more nuanced conclusions.

Higher community distress score was associated with higher PROMIS depression and anxiety T-scores at T1. It is well established that low family SES is related to poor mental health in children and adolescents [17, 33]. Among Israeli and American adults, financial strain was related to higher depression at the beginning of the COVID-19 pandemic, and worsening income loss was related to an increase in depression during the pandemic [14]. Emerging research also shows that the economic hardships of the pandemic fell most on those belonging to historically disadvantaged groups including racially minoritized groups and those with lower education levels [29]. To date, research has not examined how parental income loss during the COVID-19 pandemic has affected the mental health of youth, however, given pre-pandemic associations of parental economic status and child mental health, it is possible that pandemic-related economic hardships have impacted youth as well [17, 33]. Future research should retrospectively examine the relationships between parental income loss during the COVID-19 pandemic and youth mental health, as lessons learned from the COVID-19 pandemic may be applied to any future pandemics.

In the current sample of adolescents with high rates of pre-pandemic anxiety and depression diagnoses, the average PROMIS anxiety and depression T-scores at T1 indicated mild anxiety and moderate depression. No changes were observed between T1 and T2 in anxiety or negative affectivity. There was a statistically significant decrease in PROMIS depression T-score of 1.3 from T1 to T2, however, this is likely not a clinically significant difference as the minimally important difference for this measure is 2–3 points [35]. While some longitudinal studies found an increase in mental health symptoms from pre-pandemic to during the pandemic [12, 19], a sample of predominantly Hispanic/Latino adolescents in the Southwest U.S. with preexisting mental health problems reported a reduction in symptoms during the pandemic compared to prior. Authors hypothesized this may be related to a Hispanic/Latino cultural construct that prioritizes family, a reduction in peer-based conflict, lower academic demand, and a more natural sleep schedule [27]. Research should continue to evaluate the trajectory of mental health symptoms as the status of the pandemic changes to determine if mood symptoms will maintain current levels or begin to change.

Strengths of the present study include longitudinal assessment at two timepoints across 6 months of the COVID-19 pandemic and use of validated questionnaires. Limitations include lack of a pre-pandemic assessment timepoint and therefore inability to assess change in mental health from pre-pandemic to during the pandemic, as well as reliance on self-report rather than diagnostic assessments. There may be a survivorship bias in the sample given that there were 2947 respondents at T1 with complete data, yet only 694 respondents with complete data at T2. However, based on sensitivity analyses, no meaningful differences existed between the T2 respondents and the entire sample at T1, though it is possible that the samples differed in ways that we did not measure. Moreover, similar results emerged for all T1 models when using the full sample compared to only the respondents with complete data at T2. Another limitation that should be recognized is the large proportion of participants that reported a pre-pandemic anxiety (49%) or depression (40%) diagnosis. Adolescents with existing depression or anxiety may be more likely to respond to a mood symptom survey, therefore limiting the generalizability of our results. Further, adolescents using Facebook may have different characteristics than those not using this platform.

## Conclusion

Parents, educators, and healthcare providers should be aware of mental health symptoms endorsed by adolescents during the COVID-19 pandemic, with particular awareness that those identifying as female or other genders, and those living in distressed communities may be at a higher risk. Further, clinicians should note that as the aftereffects of the pandemic continue to be felt, anxiety and depression may remain elevated, making even more critical the need for intervention. The COVID-19 pandemic continues to evolve and impact the lives of adolescents; therefore, continued attention to the mental health of youth is imperative. Research should continue to assess mental health in adolescents and mental health screening and treatment should be an ongoing priority.

## Supplementary Information

**Additional file 1**: **Survey 1**. Full survey completed by participants.

**Additional file 2**: **Table S1**. Predictors of PROMIS Depression T-Scores at T1. Sensitivity analyses for T1 depression analyses with N = 2947.

**Additional file 3**: **Table S2**. Predictors of PROMIS Anxiety T-Scores at T1. Sensitivity analyses for T1 anxiety analyses with N = 2947.

**Additional file 4**: **Table S3**. Predictors of Negative Affect at T1. Analyses of predictors of negative affectivity at T1.

## Data Availability

Available upon request to the authors.

## Abbreviations

COVID-19: Coronavirus disease 2019
PAS: Pandemic Anxiety Scale
REDCap: Research Electronic Data Capture
T1: Timepoint 1
T2: Timepoint 2
DCI: Distressed Communities Index
PROMIS: Patient-Reported Outcomes measurement Information System
SD: Standard deviation
