# Supplementary material for: Longitudinal assessment of anxiety and depression symptoms in U.S. adolescents across six months of the coronavirus pandemic
Source: BMC Psychol. 2022 Dec 29;10:322. doi: 10.1186/s40359-022-01028-8 (PMC9798942; doi:10.1186/s40359-022-01028-8)
Supplement: Supplementary file 1 — Additional file 1: Survey 1. Full survey completed by participants. [file 40359_2022_1028_MOESM1_ESM.pdf]

Pre-Screening Survey

COVID-19 Postcard Survey
You are being asked to participate in a research study because you are a teenager between 13-19 years old.

This study plans to learn about teenagers' sleep habits, mood, and activities during COVID-19 and how they are similar or different to teenagers' sleep habits, mood and activities before COVID-19. This is important to learn about ways to help teenagers get more sleep.

If you choose to participate in this study, you will be asked to complete an anonymous online survey. You will also have the option to provide your contact e-mail address to complete the same survey in 6 months.

Possible discomforts or risks to participating in the study include feeling uncomfortable answering survey questions and breach of confidentiality. Every effort will be made to protect your privacy and confidentiality by using secured and encrypted databases for your data. The data we collect will be used for this study but may also be important for future research. Your data may be used for future research or distributed to other researchers for future study without additional consent if information that identifies you is removed from the data. Your contact information, if you choose to provide it, will not be linked to your answers. You have a choice about being in this study. You do not have to be in this study if you do not want to be. You may choose not to answer any questions on this survey at any time. The survey will take you about 15-20 minutes to complete. Your answers are very important, please answer each question as best as you can. If you have any questions, please call the research team at 720-777-4593.

You may have questions about your rights as someone in this study. If you have questions, you can call the COMIRB (the responsible Institutional Review Board). Their number is 303-724-1055.

By completing this survey, you are agreeing to participate in this research study.<br>I agree to participate in this research study:	◯ Yes<br>◯ No
How old are you?	◯ 12 or younger<br>◯ 13<br>◯ 14<br>◯ 15<br>◯ 16<br>◯ 17<br>◯ 18<br>◯ 19<br>◯ 19 or older

What grade are you in?

- ◯ 8th or lower
- ◯ 9th
- ◯ 10th
- ◯ 11th
- ◯ 12th
- ◯ Not currently enrolled in school

Where do you live?	◯ Alabama<br>◯ Alaska<br>◯ Arizona<br>◯ Arkansas<br>◯ California<br>◯ Colorado<br>◯ Connecticut<br>◯ Delaware<br>◯ Florida<br>◯ Georgia<br>◯ Hawaii<br>◯ Idaho<br>◯ Illinois<br>◯ Indiana<br>◯ Iowa<br>◯ Kansas<br>◯ Kentucky<br>◯ Louisiana<br>◯ Maine<br>◯ Maryland<br>◯ Massachusetts<br>◯ Michigan<br>◯ Minnesota<br>◯ Mississippi<br>◯ Missouri<br>◯ Montana<br>◯ Nebraska<br>◯ Nevada<br>◯ New Hampshire<br>◯ New Jersey<br>◯ New Mexico<br>◯ New York<br>◯ North Carolina<br>◯ North Dakota<br>◯ Ohio<br>◯ Oklahoma<br>◯ Oregon<br>◯ Pennsylvania<br>◯ Rhode Island<br>◯ South Carolina<br>◯ South Dakota<br>◯ Tennessee<br>◯ Texas<br>◯ Utah<br>◯ Vermont<br>◯ Virginia<br>◯ Washington<br>◯ West Virginia<br>◯ Wisconsin<br>◯ Wyoming<br>◯ Outside the United States
What is your zip code?	________________________
What type of school did you attend before the COVID-19 pandemic?	◯ In-person<br>◯ Online<br>◯ Homeschool<br>◯ Other
If other, please specify.	________________________

My parent/guardian gave me permission to participate in this research study.

◯ Yes
◯ No

DNQ Survey

Please complete the survey below.

Thank you!

Unfortunately you are not eligible to take this survey. Thank you for your interest!

Current Sleep Habits

Please answer your questions based on your sleep over the past week while you have been staying at home due to COVID-19.

In the past week, what time have you typically tried to fall asleep on weeknights?

- ◯ 8:00 pm or earlier ◯ 8:30 pm
- ◯ 9:00 pm ◯ 9:30 pm
- ◯ 10:00 pm ◯ 10:30 pm
- ◯ 11:00 pm ◯ 11:30 pm
- ◯ 12:00 am ◯ 12:30 am
- ◯ 1:00 am ◯ 1:30 am
- ◯ 2:00 am ◯ 2:30 am
- ◯ 3:00 am ◯ 3:30 am
- ◯ 4:00 am ◯ 4:30 am
- ◯ 5:00 am ◯ 5:30 am
- ◯ 6:00 am or later

In the past week, what time have you typically tried to fall asleep on the weekends?

- ◯ 8:00 pm or earlier ◯ 8:30 pm
- ◯ 9:00 pm ◯ 9:30 pm
- ◯ 10:00 pm ◯ 10:30 pm
- ◯ 11:00 pm ◯ 11:30 pm
- ◯ 12:00 am ◯ 12:30 am
- ◯ 1:00 am ◯ 1:30 am
- ◯ 2:00 am ◯ 2:30 am
- ◯ 3:00 am ◯ 3:30 am
- ◯ 4:00 am ◯ 4:30 am
- ◯ 5:00 am ◯ 5:30 am
- ◯ 6:00 am or later

In the past week, how long has it taken you to fall asleep (in minutes)?

____

In the past week, what time have you typically woken up in the morning on weekdays?

- ◯ 5:00 am or earlier ◯ 5:30 am
- ◯ 6:00 am ◯ 6:30 am
- ◯ 7:00 am ◯ 7:30 am
- ◯ 8:00 am ◯ 8:30 am
- ◯ 9:00 am ◯ 9:30 am
- ◯ 10:00 am ◯ 10:30 am
- ◯ 11:00 am ◯ 11:30 am
- ◯ 12:00 pm ◯ 12:30 pm
- ◯ 1:00 pm ◯ 1:30 pm
- ◯ 2:00 pm ◯ 2:30 pm
- ◯ 3:00 pm ◯ 3:30 pm
- ◯ 4:00 pm ◯ 4:30 pm
- ◯ 5:00 pm or later

In the past week, what time have you typically woken up in the morning on weekends?	◯ 5:00 am or earlier ◯ 5:30 am<br>◯ 6:00 am ◯ 6:30 am<br>◯ 7:00 am ◯ 7:30 am<br>◯ 8:00 am ◯ 8:30 am<br>◯ 9:00 am ◯ 9:30 am<br>◯ 10:00 am ◯ 10:30 am<br>◯ 11:00 am ◯ 11:30 am<br>◯ 12:00 pm ◯ 12:30 pm<br>◯ 1:00 pm ◯ 1:30 pm<br>◯ 2:00 pm ◯ 2:30 pm<br>◯ 3:00 pm ◯ 3:30 pm<br>◯ 4:00 pm ◯ 4:30 pm<br>◯ 5:00 pm or later
In the past week, how often have you taken naps during the daytime?	◯ Never (0 days)<br>◯ Once in a while (1-2 days)<br>◯ Sometimes (2-3 days)<br>◯ Quite Often (4-5 days)<br>◯ Frequently (5-6 days)<br>◯ Always (7 days)
How long do you nap?	◯ 0-30 minutes<br>◯ 30-60 minutes<br>◯ 60+ minutes<br>◯ I do not nap
How many nights have your parents/caregivers told you to go to sleep in the past week?	◯ Never (0 nights)<br>◯ Once in a while (1-2 nights)<br>◯ Sometimes (2-3 nights)<br>◯ Quite Often (4-5 nights)<br>◯ Frequently (5-6 nights)<br>◯ Always (7 nights)
How often have your parents/caregivers woken you up in the morning in the past week?	◯ Never (0 mornings)<br>◯ Once in a while (1-2 mornings)<br>◯ Sometimes (2-3 mornings)<br>◯ Quite Often (4-5 mornings)<br>◯ Frequently (5-6 mornings)<br>◯ Always (7 mornings)
In the past week, have you used any medications/supplements to help with sleep? (Mark all that apply)	☐ Melatonin<br>☐ Tylenol PM<br>☐ Nyquil or ZzzQuil<br>☐ Benadryl<br>☐ Clonidine<br>☐ Trazadone<br>☐ Quetiapine (Seroquel)<br>☐ Gabapentin (Neurontin)<br>☐ Eszopicline (Lunesta)<br>☐ Zolpidem (Ambien)<br>☐ Zaleplon (Sonata)<br>☐ Other: ________
If other, please specify.	__________________________________

How often did you use one of these medications/supplements in the past week?

- ◯ Never (0 nights)
- ◯ Once in a while (1-2 nights)
- ◯ Sometimes (2-3 nights)
- ◯ Quite Often (4-5 nights)
- ◯ Frequently (5-6 nights)
- ◯ Always (7 nights)

Pediatric Insomnia Severity Index Pre-COVID

Please answer the following questions based on your sleep over a typical week before COVID-19.

1) It took me longer than 30 minutes to fall asleep after going to bed.
- ◯ Never (0 nights)
- ◯ Once in a while (1-2 nights)
- ◯ Sometimes (2-3 nights)
- ◯ Quite Often (4-5 nights)
- ◯ Frequently (5-6 nights)
- ◯ Always (7 nights)

2) In general I had trouble going to sleep.
- ◯ Never (0 nights)
- ◯ Once in a while (1-2 nights)
- ◯ Sometimes (2-3 nights)
- ◯ Quite Often (4-5 nights)
- ◯ Frequently (5-6 nights)
- ◯ Always (7 nights)

3) During the night I woke up more than once.
- ◯ Never (0 days)
- ◯ Once in a while (1-2 days)
- ◯ Sometimes (2-3 days)
- ◯ Quite Often (4-5 days)
- ◯ Frequently (5-6 days)
- ◯ Always (7 days)

4) After waking up during the night I had trouble going back to sleep.
- ◯ Never (0 days)
- ◯ Once in a while (1-2 days)
- ◯ Sometimes (2-3 days)
- ◯ Quite Often (4-5 days)
- ◯ Frequently (5-6 days)
- ◯ Always (7 days)

5) I felt sleepy during the day.
- ◯ Never (0 days)
- ◯ Once in a while (1-2 days)
- ◯ Sometimes (2-3 days)
- ◯ Quite Often (4-5 days)
- ◯ Frequently (5-6 days)
- ◯ Always (7 days)

6) How many hours of sleep did you get on most nights?
- ◯ >9
- ◯ 8-9
- ◯ 7-8
- ◯ 6-7
- ◯ 5-6
- ◯ Less than 5

Current Activities

Please answer the following questions based on your response since being at home due to COVID-19.

Are you currently attending in-person classes at school?

◯ Yes
◯ No
◯ N/A (online or homeschool before COVID-19)

Are you doing online or distance schooling?

◯ Yes
◯ No

Does your distance or online schooling require you start at a set time each morning?

◯ Yes
◯ No

What time do you have to start online school each morning?

◯ 6:00 am or earlier
◯ 6:30 am
◯ 7:00 am
◯ 7:30 am
◯ 8:00 am
◯ 8:30 am
◯ 9:00 am
◯ 9:30 am
◯ 10:00 am
◯ 10:30 am
◯ 11:00 am
◯ 11:30 am
◯ 12:00 pm
◯ 12:30 pm
◯ 1:00 pm
◯ 1:30 pm
◯ 2:00 pm
◯ 2:30 pm
◯ 3:00 pm
◯ 3:30 pm
◯ 4:00 pm
◯ 4:30 pm
◯ 5:00 pm or later

Compared to your school start time before COVID-19 is this new time:

◯ Earlier
◯ Later
◯ The same

Have you changed the amount of time you spend on activities SINCE THE START OF COVID-19? For each activity, please indicate if you are spending more time, less time, or the same amount of time SINCE THE START OF COVID-19.

	More Time	Less Time	Same Amount	I don't do this
Technology (e.g., social media, playing video/computer games, watching TV/movies)	◯	◯	◯	◯

Physical activity (e.g., dancing, riding a bike)	◯	◯	◯	◯
Spending time outside (e.g., spending time outside, walking, sitting on the porch)	◯	◯	◯	◯
Extracurricular activities (e.g., sports, school clubs, church, internship through technology or in person)	◯	◯	◯	◯
Social interaction (e.g., spending time with friends or family through technology or in person)	◯	◯	◯	◯
Working at my job	◯	◯	◯	◯
Schoolwork	◯	◯	◯	◯

Since being at home due to COVID-19, when do you currently do most of your schoolwork?

- ◯ In the morning (before noon)
- ◯ In the afternoon (12-5pm)
- ◯ In the evening (6-9pm)
- ◯ At night (After 9pm)
- ◯ Do not have schoolwork

Since being at home due to COVID-19, how much time do you spend in your bed during the day while awake (in hours)? This refers to doing an activity in your bed, such as using your phone or doing schoolwork.

- ◯ Less than 30 minutes
- ◯ 30 minutes to 1 hour
- ◯ 1-2 hours
- ◯ 3-4 hours
- ◯ 5-6 hours
- ◯ 6-7 hours
- ◯ 8-9 hours
- ◯ 10-11 hours
- ◯ 12+ hours

Since being at home due to COVID-19, what activities are you doing in your bed? Please mark all that apply.

- ☐ Using your phone
- ☐ Social media
- ☐ Playing video/computer games
- ☐ Watching TV/movies
- ☐ Schoolwork/homework
- ☐ Reading
- ☐ Other: ________
- ☐ I don't do activities in my bed

If other, please specify.

____

Changes in Sleep Habits

Please complete the following questions based on a typical week of sleep before COVID-19 when you were on your regular schedule and attending school as usual.

Before COVID-19, what time did you typically try to fall asleep on weeknights?

◯ 8:00 pm or earlier ◯ 8:30 pm
◯ 9:00 pm ◯ 9:30 pm
◯ 10:00 pm ◯ 10:30 pm
◯ 11:00 pm ◯ 11:30 pm
◯ 12:00 am ◯ 12:30 am
◯ 1:00 am ◯ 1:30 am
◯ 2:00 am ◯ 2:30 am
◯ 3:00 am ◯ 3:30 am
◯ 4:00 am ◯ 4:30 am
◯ 5:00 am ◯ 5:30 am
◯ 6:00 am or later

Before COVID-19, what time did you typically try to fall asleep on the weekends?

◯ 8:00 pm or earlier ◯ 8:30 pm
◯ 9:00 pm ◯ 9:30 pm
◯ 10:00 pm ◯ 10:30 pm
◯ 11:00 pm ◯ 11:30 pm
◯ 12:00 am ◯ 12:30 am
◯ 1:00 am ◯ 1:30 am
◯ 2:00 am ◯ 2:30 am
◯ 3:00 am ◯ 3:30 am
◯ 4:00 am ◯ 4:30 am
◯ 5:00 am ◯ 5:30 am
◯ 6:00 am or later

Before COVID-19, how long did it take you to fall asleep (in minutes)?

____

Before COVID-19, what time did you usually wake up on school days?

◯ 5:00 am or earlier ◯ 5:30 am
◯ 6:00 am ◯ 6:30 am
◯ 7:00 am ◯ 7:30 am
◯ 8:00 am ◯ 8:30 am
◯ 9:00 am ◯ 9:30 am
◯ 10:00 am ◯ 10:30 am
◯ 11:00 am ◯ 11:30 am
◯ 12:00 pm ◯ 12:30 pm
◯ 1:00 pm ◯ 1:30 pm
◯ 2:00 pm ◯ 2:30 pm
◯ 3:00 pm ◯ 3:30 pm
◯ 4:00 pm ◯ 4:30 pm
◯ 5:00 pm or later

Before COVID-19, what time did you usually wake up on weekends?	◯ 5:00 am or earlier ◯ 5:30 am<br>◯ 6:00 am ◯ 6:30 am<br>◯ 7:00 am ◯ 7:30 am<br>◯ 8:00 am ◯ 8:30 am<br>◯ 9:00 am ◯ 9:30 am<br>◯ 10:00 am ◯ 10:30 am<br>◯ 11:00 am ◯ 11:30 am<br>◯ 12:00 pm ◯ 12:30 pm<br>◯ 1:00 pm ◯ 1:30 pm<br>◯ 2:00 pm ◯ 2:30 pm<br>◯ 3:00 pm ◯ 3:30 pm<br>◯ 4:00 pm ◯ 4:30 pm<br>◯ 5:00 pm or later
Before COVID-19, in a typical week, did you previously take naps during the daytime?	◯ Never (0 days)<br>◯ Once in a while (1-2 days)<br>◯ Sometimes (2-3 days)<br>◯ Quite Often (4-5 days)<br>◯ Frequently (5-6 days)<br>◯ Always (7 days)
Before COVID-19, in a typical week, how long did you nap?	◯ 0-30 minutes<br>◯ 30-60 minutes<br>◯ 60+ minutes<br>◯ I did not nap
Before COVID-19, how often did your parents/caregivers tell you to go to sleep in a typical week?	◯ Never (0 nights)<br>◯ Once in a while (1-2 nights)<br>◯ Sometimes (2-3 nights)<br>◯ Quite Often (4-5 nights)<br>◯ Frequently (5-6 nights)<br>◯ Always (7 nights)
Before COVID-19, how often did your parents/caregivers wake you up in the morning in a typical week?	◯ Never (0 mornings)<br>◯ Once in a while (1-2 mornings)<br>◯ Sometimes (2-3 mornings)<br>◯ Quite Often (4-5 mornings)<br>◯ Frequently (5-6 mornings)<br>◯ Always (7 mornings)
Before COVID-19, in a typical week, did you previously use any medications/supplements to help with sleep? (Mark all that apply)	☐ Melatonin<br>☐ Tylenol PM<br>☐ Nyquil or ZzzQuil<br>☐ Benadryl<br>☐ Clonidine<br>☐ Trazadone<br>☐ Quetiapine (Seroquel)<br>☐ Gabapentin (Neurontin)<br>☐ Eszopicline (Lunesta)<br>☐ Zolpidem (Ambien)<br>☐ Zaleplon (Sonata)<br>☐ Other: _______
If other, please specify.	________________________________

Before COVID-19, how often did you use one of these medications/supplements in a typical week?

- ◯ Never (0 nights)
- ◯ Once in a while (1-2 nights)
- ◯ Sometimes (2-3 nights)
- ◯ Quite Often (4-5 nights)
- ◯ Frequently (5-6 nights)
- ◯ Always (7 nights)

Pediatric Insomnia Severity Index Current

Please answer the following questions based on your sleep over the past week.

1) In general I have trouble going to sleep.
- ◯ Never (0 nights)
- ◯ Once in a while (1-2 nights)
- ◯ Sometimes (2-3 nights)
- ◯ Quite Often (4-5 nights)
- ◯ Frequently (5-6 nights)
- ◯ Always (7 nights)

2) It takes me longer than 30 minutes to fall asleep after going to bed.
- ◯ Never (0 nights)
- ◯ Once in a while (1-2 nights)
- ◯ Sometimes (2-3 nights)
- ◯ Quite Often (4-5 nights)
- ◯ Frequently (5-6 nights)
- ◯ Always (7 nights)

3) During the night I wake up more than once.
- ◯ Never (0 days)
- ◯ Once in a while (1-2 days)
- ◯ Sometimes (2-3 days)
- ◯ Quite Often (4-5 days)
- ◯ Frequently (5-6 days)
- ◯ Always (7 days)

4) After waking up during the night I have trouble going back to sleep.
- ◯ Never (0 days)
- ◯ Once in a while (1-2 days)
- ◯ Sometimes (2-3 days)
- ◯ Quite Often (4-5 days)
- ◯ Frequently (5-6 days)
- ◯ Always (7 days)

5) I feel sleepy during the day.
- ◯ Never (0 days)
- ◯ Once in a while (1-2 days)
- ◯ Sometimes (2-3 days)
- ◯ Quite Often (4-5 days)
- ◯ Frequently (5-6 days)
- ◯ Always (7 days)

6) How many hours of sleep do you get on most nights?
- ◯ >9
- ◯ 8-9
- ◯ 7-8
- ◯ 6-7
- ◯ 5-6
- ◯ Less than 5

Activities Pre-COVID

Please answer the following questions based on your response before COVID-19.	
Before COVID-19, when did you do most of your schoolwork?	◯ In the morning (before noon)<br>◯ In the afternoon (12-5pm)<br>◯ In the evening (6-9pm)<br>◯ At night (After 9pm)<br>◯ Did not have schoolwork
Before COVID-19, how much time did you spend in your bed during the day while awake (in hours)?	◯ Less than 30 minutes<br>◯ 30 minutes to 1 hour<br>◯ 1-2 hours<br>◯ 3-4 hours<br>◯ 5-6 hours<br>◯ 6-7 hours<br>◯ 8-9 hours<br>◯ 10-11 hours<br>◯ 12+ hours
Before COVID-19, what activities did you do in your bed? Please mark all that apply.	☐ Using your phone<br>☐ Social media<br>☐ Playing video/computer games<br>☐ Watching TV/movies<br>☐ Schoolwork/homework<br>☐ Reading<br>☐ Other: _______
If other, please specify.	______________________________

Mood

Please respond to each question or statement by marking one answer per row.
In the past 7 days...

		Never	Almost Never	Sometimes	Often	Almost Always
1)	I've worried about getting sick or catching COVID-19.	○	○	○	○	○
2)	I've worried about the health of my friends and family or I've worried about my friends and family getting sick.	○	○	○	○	○
3)	Worries or thoughts about COVID-19 have made it hard to sleep at night.	○	○	○	○	○
4)	Worries or thoughts make it hard to sleep at night.	○	○	○	○	○

Please respond to each question or statement by marking one answer per row.

In the past 7 days...

		Never	Almost Never	Sometimes	Often	Almost Always
5)	I could not stop feeling sad.	○	○	○	○	○
6)	I felt alone.	○	○	○	○	○
7)	I felt everything in my life went wrong.	○	○	○	○	○
8)	I felt like I couldn't do anything right.	○	○	○	○	○
9)	I felt lonely.	○	○	○	○	○
10)	I felt sad.	○	○	○	○	○
11)	I felt unhappy.	○	○	○	○	○
12)	It was hard for me to have fun.	○	○	○	○	○

Please respond to each question or statement by marking one answer per row.

In the past 7 days....

		Never	Almost Never	Sometimes	Often	Almost Always
13)	I felt like something awful might happen.	○	○	○	○	○
14)	I felt nervous.	○	○	○	○	○
15)	I felt scared.	○	○	○	○	○
16)	I felt worried.	○	○	○	○	○

17)

	I worried when I was at home.	◯	◯	◯	◯	◯
18)	I got scared really easy.	◯	◯	◯	◯	◯
19)	I worried about what could happen to me.	◯	◯	◯	◯	◯
20)	I worried when I went to bed at night.	◯	◯	◯	◯	◯

Morningness/Eveningness Scale for Children

Please answer the following questions based on a typical week pre-COVID-19 when you were attending school as usual.

1) Imagine: School is cancelled! You can get up whenever you want to. When would you get out of bed? Between...
- ◯ 5:00-6:30 am
- ◯ 6:30 and 7:45 am
- ◯ 7:45 and 9:45 am
- ◯ 11:00 am and noon

2) Is it easy for you to get up in the morning?
- ◯ No way!
- ◯ Sort of
- ◯ Pretty easy
- ◯ It's a cinch

3) Gym class is set for 7:00 in the morning. How do you think you'll do?
- ◯ My best!
- ◯ Okay
- ◯ Worse than usual
- ◯ Awful

4) The bad news: You have to take a two-hour test. The good news: You can take it when you think you'll do your best. What time is that?
- ◯ 8:00 to 10:00 am
- ◯ 11:00 am to 1:00 pm
- ◯ 3:00 to 5:00 pm
- ◯ 7:00 to 9:00 pm

5) When do you have the most energy to do your favorite things?
- ◯ Morning! I'm tired in the evening
- ◯ Morning more than evening
- ◯ Evening more than morning
- ◯ Evening! I'm tired in the morning

6) Guess what? Your parents have decided to let you set your own bedtime. What time would you pick?
- ◯ 8:00 and 9:00 pm
- ◯ 9:00 and 10:15 pm
- ◯ 10:15 pm and 12:30 am
- ◯ 12:30 and 1:45 am
- ◯ 1:45 and 3:00 am

7) How alert are you in the first half hour you're up?
- ◯ Out of it
- ◯ A little dazed
- ◯ Okay
- ◯ Ready to take on the world

8) When does your body start to tell you it's time for bed (even if you ignore it)? Between...
- ◯ 8:00 and 9:00 pm
- ◯ 9:00 and 10:15 pm
- ◯ 10:15 pm and 12:30 am
- ◯ 12:30 and 1:45 am
- ◯ 1:45 and 3:00 am

9) Say you had to get up at 6:00 am every morning: What would it be like?
- ◯ Awful!
- ◯ Not so great
- ◯ Okay (if I have to)
- ◯ Fine, no problem

10) When you wake up in the morning, how long does it take for you to be totally "with it"?
- ◯ 0 to 10 minutes
- ◯ 11 to 20 minutes
- ◯ 21 to 40 minutes
- ◯ More than 40 minutes

Demographics

What is your gender?	◯ Male<br>◯ Female<br>◯ Other: _______<br>◯ Nonbinary
If other, please specify.	__________________________________
What is your race?	☐ White<br>☐ Black<br>☐ Asian<br>☐ Hispanic<br>☐ American Indian or Alaska Native<br>☐ Native Hawaiian or Pacific Islander<br>☐ Other: _______<br>☐ Choose not to answer
If other, please specify.	__________________________________
Has a healthcare provider (e.g., doctor, nurse, psychologist, counselor) ever told you that you have one of the following problems? Please select all that apply.	☐ Insomnia<br>☐ Obstructive Sleep Apnea or Sleep Disordered Breathing<br>☐ Delayed Sleep Phase (Circadian Rhythm Disorder)<br>☐ Narcolepsy<br>☐ Restless leg syndrome or periodic limb movement disorder<br>☐ Central Sleep Apnea<br>☐ Excessive daytime sleepiness<br>☐ Depression<br>☐ Anxiety<br>☐ ADHD<br>☐ Other mood disorder<br>☐ Autism<br>☐ Allergies<br>☐ Asthma<br>☐ GERD/Reflux<br>☐ Overweight or obese<br>☐ Heart disease<br>☐ High blood pressure<br>☐ Thyroid problems<br>☐ Sickle Cell disease<br>☐ Eczema/ atopic dermatitis<br>☐ Diabetes<br>☐ Chronic pain (e.g., headache; stomach; nerve)<br>☐ Other: __________<br>☐ None of these
If other, please specify.	__________________________________
Are you currently experiencing symptoms (e.g., cough, difficulty breathing, fever) of COVID-19?	◯ Yes<br>◯ No
Have you been diagnosed with COVID-19?	◯ Yes<br>◯ No

Is your state/city/region on stay at home orders (e.g., rules about what activities you are allowed to do outside the home)?	◯ Yes<br>◯ No<br>◯ Unsure
Please enter your e-mail address, if you would like to be contacted to complete a follow-up survey of the same questions about your sleep in 6 months. Your e-mail address will only be used to send you a link to complete a follow-up survey.	__________________________________
