# Supplementary material for: Longitudinal assessment of anxiety and depression symptoms in U.S. adolescents across six months of the coronavirus pandemic
Source: BMC Psychol. 2022 Dec 29;10:322. doi: 10.1186/s40359-022-01028-8 (PMC9798942; doi:10.1186/s40359-022-01028-8)
Supplement: Supplementary file 2 — Additional file 2: Table S1. Predictors of PROMIS Depression T-Scores at T1. Sensitivity analyses for T1 depression analyses with N = 2947. [file 40359_2022_1028_MOESM2_ESM.docx]

**Sensitivity Analysis for T1 Analyses with N=2947**

**Supplementary Table 1:** Predictors of PROMIS Depression T-Scores at T1

|  | **PROMIS Depression T-score at T1 with follow-up data (N=694)** | | | **PROMIS Depression T-score at T1 without follow-up data (N=2947)** | | |
| --- | --- | --- | --- | --- | --- | --- |
| *Predictors* | *Estimates* | *CI* | *p* | *Estimates* | *CI* | *p* |
| (Intercept) | 53.11 | 40.28, 65.95 | **<0.001** | 63.45 | 57.27, 69.63 | **<0.001** |
| Gender [Female vs. Male] | 4.67 | 2.82, 6.52 | **<0.001** | 5.03 | 4.12, 5.94 | **<0.001** |
| Gender [Other vs. Male] | 6.44 | 2.89, 9.99 | **<0.001** | 8.01 | 6.16, 9.87 | **<0.001** |
| Race [White vs. Non-White] | 0.58 | -1.84, 3.01 | 0.637 | 0.23 | -0.94, 1.41 | 0.698 |
| Ethnicity [Hispanic vs. Non-Hispanic] | 0.89 | -2.66, 4.44 | 0.623 | -0.04 | -1.86, 1.78 | 0.964 |
| Age (years) | 0.41 | -0.36, 1.17 | 0.296 | -0.21 | -0.57, 0.16 | 0.275 |
| Distress Score | 0.04 | 0.01, 0.07 | **0.003** | 0.02 | 0.01, 0.04 | **0.003** |
