# Supplementary material for: Longitudinal assessment of anxiety and depression symptoms in U.S. adolescents across six months of the coronavirus pandemic
Source: BMC Psychol. 2022 Dec 29;10:322. doi: 10.1186/s40359-022-01028-8 (PMC9798942; doi:10.1186/s40359-022-01028-8)
Supplement: Supplementary file 3 — Additional file 3: Table S2. Predictors of PROMIS Anxiety T-Scores at T1. Sensitivity analyses for T1 anxiety analyses with N = 2947. [file 40359_2022_1028_MOESM3_ESM.docx]

**Sensitivity Analysis for T1 Analyses with N=2947**

**Supplementary Table 2:** Predictors of PROMIS Anxiety T-Scores at T1

|  | **PROMIS Anxiety T-score at T1 with follow-up data (N=694)** | | | **PROMIS Anxiety T-score at T1 without follow-up data (N=2947)** | | |
| --- | --- | --- | --- | --- | --- | --- |
| *Predictors* | *Estimates* | *CI* | *p* | *Estimates* | *CI* | *p* |
| (Intercept) | 47.65 | 32.23, 63.07 | **<0.001** | 56.94 | 49.73, 64.15 | **<0.001** |
| Gender [Female vs. Male] | 6.02 | 3.80, 8.24 | **<0.001** | 6.72 | 5.65, 7.78 | **<0.001** |
| Gender [Other vs. Male] | 6.71 | 2.44, 10.97 | **0.002** | 10.44 | 8.28, 12.60 | **<0.001** |
| Race [White vs. Non-White] | 0.90 | -2.01, 3.81 | 0.544 | 0.23 | -1.14, 1.60 | 0.745 |
| Ethnicity [Hispanic vs. Non-Hispanic] | 0.61 | -3.66, 4.88 | 0.779 | 1.08 | -1.04, 3.20 | 0.317 |
| Age (years) | 0.24 | -0.67, 1.16 | 0.602 | -0.29 | -0.72, 0.14 | 0.181 |
| Distress Score | 0.06 | 0.02, 0.09 | **0.001** | 0.02 | 0.01, 0.04 | **0.005** |
