# Supplementary material for: Longitudinal assessment of anxiety and depression symptoms in U.S. adolescents across six months of the coronavirus pandemic
Source: BMC Psychol. 2022 Dec 29;10:322. doi: 10.1186/s40359-022-01028-8 (PMC9798942; doi:10.1186/s40359-022-01028-8)
Supplement: Supplementary file 4 — Additional file 4: Table S3. Predictors of Negative Affect at T1. Analyses of predictors of negative affectivity at T1. [file 40359_2022_1028_MOESM4_ESM.docx]

**Supplementary Table 3:** Predictors of Negative Affect at T1

|  | **Negative Affect at T1 with follow-up data (N=694)** | | | **Negative Affect at T1 without follow-up data (N=2947)** | | |
| --- | --- | --- | --- | --- | --- | --- |
| *Predictors* | *Odds Ratios* | *CI* | *p* | *Odds Ratios* | *CI* | *p* |
| (Intercept) | 0.31 | 0.03, 3.48 | 0.344 | 0.68 | 0.22, 2.10 | 0.501 |
| Gender [Female vs. Male] | 2.13 | 1.49, 3.07 | **<0.001** | 2.43 | 2.05, 2.89 | **<0.001** |
| Gender [Other vs. Male] | 1.80 | 0.92, 3.52 | 0.084 | 3.61 | 2.57, 5.10 | **<0.001** |
| Race [White vs. Non-White] | 1.04 | 0.66, 1.65 | 0.855 | 1.05 | 0.84, 1.30 | 0.686 |
| Ethnicity [Hispanic vs. Non-Hispanic] | 1.43 | 0.73, 2.82 | 0.295 | 1.21 | 0.87, 1.68 | 0.267 |
| Age (years) | 1.01 | 0.87, 1.16 | 0.906 | 0.96 | 0.90, 1.03 | 0.255 |
| Distress Score | 1.01 | 1.00, 1.01 | **0.017** | 1.00 | 1.00, 1.01 | 0.076 |
